# Platelet-Activating Factor Induces Th17 Cell Differentiation

**DOI:** 10.1155/2011/913802

**Published:** 2011-10-13

**Authors:** Anne-Marie Drolet, Maryse Thivierge, Sylvie Turcotte, Dominique Hanna, Bruno Maynard, Jana Stankovà, Marek Rola-Pleszczynski

**Affiliations:** ^1^Immunology Division, Department of Pediatrics, Faculty of Medicine and Health Sciences, Université de Sherbrooke, Sherbrooke, QC, Canada J1H 5N4; ^2^Dermatology Division, Department of Medicine, Faculty of Medicine and Health Sciences, Université de Sherbrooke, Sherbrooke, QC, Canada J1H 5N4

## Abstract

Th17 cells have been implicated in a number of inflammatory and autoimmune diseases. The phospholipid mediator platelet-activating factor (PAF) is found in increased concentrations in inflammatory lesions and has been shown to induce IL-6 production. We investigated whether PAF could affect the development of Th17 cells. Picomolar concentrations of PAF induced IL-23, IL-6, and IL-1**β** expression in monocyte-derived Langerhans cells (LCs) and in keratinocytes. Moreover, when LC were pretreated with PAF and then cocultured with anti-CD3- and anti-CD28-activated T cells, the latter developed a Th17 phenotype, with a significant increase in the expression of the transcriptional regulator ROR**γ**t and enhanced expression of IL-17, IL-21, and IL-22. PAF-induced Th17 development was prevented by the PAF receptor antagonist WEB2086 and by neutralizing antibodies to IL-23 and IL-6R. This may constitute a previously unknown stimulus for the development and persistence of inflammatory processes that could be amenable to pharmacologic intervention.

## 1. Introduction

A unique subset of interleukin (IL)-17-producing CD4^+^ T helper (Th17) cells, distinct from the well-known Th1 and Th2 cells, was recently identified. The differentiation and persistence of Th17 cells was shown to be dependent on the presence of selected cytokines that include, in humans, IL-1*β*, IL-6 [1], and IL-23 [2–4], as well as TGF*β* [5]. Th17 cells secrete a range of cytokines, including IL-17A, IL-17F, IL-21, IL-22, TNF*α*, and IL-6, which have both overlapping and distinct roles in host defense and inflammation [4, 6]. Retinoic orphan receptor-gamma T (ROR*γ*t)^3^ and, in humans, its orthologue RORC2 were identified as markers of Th17 cells and shown to be master regulatory transcription factors required for Th17 development [7, 8]. IL-17 appears to play essential roles not only in host defenses against various pathogens, but also in the pathogenesis of chronic inflammatory disorders and in many autoimmune diseases, including multiple sclerosis, inflammatory bowel disease, asthma, and psoriasis [9–15].

Psoriasis is a chronic inflammatory skin disorder characterized by hyperplasia of the epidermis, infiltration of leukocytes into both the dermis and the epidermis, as well as dilatation and growth of blood vessels. Until recently, psoriasis has been considered mainly to be a Th1-driven autoimmune inflammatory disease, but recent findings have clearly revealed a role for IL-23 and Th17 cells. In support of this, an increased number of Th17 cells has been identified in the dermis and epidermis of psoriatic skin compared with normal skin [16], and these cells are activated based on increased IL-17A, IL-17C, IL-17F, and IL-22 expression. Moreover, expression of IL-17A, IL-17F, IL-26, CCL20, and ROR*γ*t, all Th17 markers, were shown to be enhanced in psoriatic skin [2, 17]. IL-23 was also reported to be highly expressed in lesional psoriatic skin and to be produced by keratinocytes, Langerhans cells (LC), dermal dendritic cells and macrophages [18, 19]. Finally, resolution of psoriatic lesions has been achieved with the use of several kinds of immune modulators that block the IL-23/Th17 [20].

Platelet-activating factor (PAF) is a potent phospholipid inflammatory mediator that is released early in inflammation by a variety of cell types. PAF acts largely by binding to its receptor (PAFR), a G-protein-coupled receptor found on most cells, including platelets, monocytes, mast cells, granulocytes, B lymphocytes, dendritic cells, and keratinocytes [21–24]. PAF is a known regulator of transcription and has been shown to upregulate the secretion of a variety of cytokines, including IL-1, IL-6, and TNF-*α* [25, 26]. PAF has been implicated in the pathogenesis of asthma and other allergic conditions, in inflammatory bowel disease, rheumatoid arthritis, multiple sclerosis, endotoxic shock, and dermal inflammation [23, 27, 28]. Several observations suggested a role for PAF in psoriasis. Hence, it was reported that PAF plasma levels were elevated in patients with psoriasis and that lesional psoriatic skin contains substantial amounts of this mediator [29, 30]. Histological analysis has shown greater PAFR staining in the epidermis of psoriasis patients compared to controls [31]. A thickened skin with increased proliferation of epidermal keratinocytes, as is seen in psoriasis, was observed in transgenic mice which overexpress PAFR [32].

In the current study, we examined the potential for PAF to induce Th17 development through activation of LC and production of IL-6 and IL-23, in a model of LC-T cell coculture.

## 2. Materials and Methods

### 2.1. Generation and Isolation of Monocyte-Derived Langerhans Cells

Monocyte-derived LC were generated from human peripheral blood mononuclear leukocytes (PBML) obtained from normal donors following informed consent in accordance with an Internal Review Board-approved protocol, in conformity with the Declaration of Helsinki. Blood monocytes were purified by density gradient centrifugation on Ficoll-Paque (GE healthcare, Piscataway, NJ, USA), followed by plastic adherence, and were cultured for 5-6 days in 6-well tissue culture plates (Becton Dickinson Labware, Franklin Lakes, NJ, USA) at 2 × 10^6^/mL in RPMI 1640 medium supplemented with 10% (v/v) FBS (PAA Laboratories), rhGM-CSF (20 ng/mL), rhIL-4 (20 ng/mL) and rh-TGF-*β* (10 ng/mL) (Peprotech, Rocky Hill, NJ, USA) at 37°C in a humidified 5% CO_2_ incubator. On day 3, fresh medium supplemented with the above mentioned cytokines was added. After 5 days of culture, the outcoming population consisted of typical immature LC to which half-strength concentrations of above mentioned cytokines were added. These LC expressed low levels of CD86, and were negative for CD83 (BD Pharmingen, Mississauga, ON, Canada). They were routinely tested for langerin (Beckman Coulter, Marseille, France) and E-cadherin (R&D Systems, Minneapolis, Minn, USA) expression, which exceeded 80% and 75%, respectively.

### 2.2. Isolation of CD4^+^ T Cells

CD4^+^ T cells were purified from whole blood lymphocytes by depletion of contaminating cells using a “Human CD4^+^ T cell enrichment kit” (Stem Cell technologies, Vancouver, BC, Canada) following the manufacturer's instructions. Purity was greater than 98%. CD4^+^ T cells at 0.5 × 10^6^ cells/mL in RPMI 1640 10% FBS were then incubated for 5 days with a combination of anti-CD3 (2 *μ*g/mL), immobilized on microplates, and soluble anti-CD28 (1 *μ*g/mL) antibodies. The treatment was effective in inducing T cell blastogenesis.

### 2.3. Coculture of PAF-Stimulated MoLC with T Cells

T cells were plated at 1 × 10^5^ cells/well and either stimulated alone with a combination of IL-1*β* + IL-6 + IL-23 (Peprotech, Rocky Hill, NJ and Alexis Biochemicals, San Diego, Calif, USA) for 5 days or cocultured with 2.5 × 10^4^ autologous LC in the absence or presence of graded concentrations of PAF (10^−12^ to 10^−7^ M) (octadecyl-PAF, Cayman, Ann Arbor, Mich, USA). When indicated, neutralizing Ab for IL-6R, IL-15 or IL-23p19 (R&D Systems) were used at 0.4 *μ*g/mL, 0.5 *μ*g/mL and 0.8 *μ*g/mL, respectively. Cultures were used after 5 days for cytometry and PCR analysis. When indicated, inhibitors of Jak2 (AG490), EGFR (EGFR Inhibitor), NF-*κ*B (NF-*κ*B Activation Inhibitor) or STAT3 (STAT3 Inhibitor Peptide), all from EMD Biosciences, San Diego, Calif, USA, were at 10 *μ*M, 20 *μ*M, 20 *μ*M and 400 *μ*M, respectively.

### 2.4. Keratinocyte Cultures

The A431 human keratinocytic squamous cell carcinoma cell line, was obtained from the American Type Culture Collection and cultured at 37°C in high-glucose DMEM (Gibco BRL, Grand Island, NY, USA) supplemented with 10% fetal bovine serum (FBS) (PAA Laboratories, Etobicoke, ON, Canada).

Normal human neonatal foreskin epidermal keratinocytes (NHEK cells) were obtained from Lonza (Walkersville, Md, USA). NHEK cells were grown in serum-free medium, KGM-2 (Lonza), containing 0.09 mmol/L CaCl_2_, 0.5 mg/mL hydrocortisone, 0.1 ng/mL recombinant human epidermal growth factor (EGF), 5 ng/mL insulin, 0.4% v/v bovine pituitary extract, 50 mg/mL gentamycin and 0.05 mg/mL amphotericin B, in an atmosphere of 95% air and 5% CO_2_ at 37°C. NHEK cells were used in the proliferative phase at 70%–80% confluency.

### 2.5. Flow Cytometry Analysis

For the last 3 h of coculture, cells were stimulated with 25 ng/mL PMA (Sigma-Aldrich, Oakville, ON, Canada) and 1 *μ*g/mL ionomycin (EMD chemicals, La Jolla, Calif, USA) in the presence of 2 mM monensin (BD Biosciences, Mississauga, ON, Canada) for assessment of intracellular IL-17A. Cells were then washed in Fix/Perm solution (eBioscience, San Diego, Calif, USA) according to the manufacturer's instructions and stained for CD4 (BD biosciences) and intracellular IL-17A or ROR*γ*t (eBioscience) for 30 minutes. After washing, cells were analyzed on a FACSCalibur flow cytometer using the CellQuestPro software.

### 2.6. RNA Isolation and Real-Time Quantitative PCR

RNA was obtained using Trizol reagent (Invitrogen, Burlington, ON, Canada) according to the manufacturer's instructions. After total RNA purification with Rneasy kit (Qiagen, Mississauga, ON, Canada), 1.0 *μ*g of RNA was converted to cDNA with oligo dT (Fermentas, Burlington, ON, Canada) and reverse transcriptase (M-MLV; Promega, Madison, WI, USA) in a volume of 20 *μ*L. GAPDH, IL-6, IL-17A, IL-21, IL-22, IL-23p19, PAF receptor (PAFR) and RORC2 expression were measured using real-time PCR performed on a Rotor-Gene 3000 (Corbett Research, Kirkland, QC, Canada). The following oligonucleotide primer sets were obtained from IDT (Coralville, Ind, USA): human GAPDH: forward, 5-GAT GAC ATC AAG AAG GTG GTG AA-3 and reverse, 5-GTC TTA CTC CTT GGA GGC CAT GT-3; human IL-6: forward, 5-GTG TGA AAG CAG CAA AGA GGC-3 and reverse, 5-CTG GAG GTA CTC TAG GTA TAC-3; human IL-17A: forward, 5-CTA CAA CCG ATC CAC CTC AC-3 and reverse, 5-CCA CGG ACA CCA GTA TCT TC-3; human IL-21: forward, 5-GCA ACA TGG AGA GGA TTG TC-3 and reverse, 5-CTG AAA GCA GGA AAA AGC TG-3; human IL-22: forward, 5-CAC AGA CGT TCG TCT CAT TG-3 and reverse, 5-AGC TTT TGC ACA TTC CTC TG-3; human IL-23p19: forward, 5-GAT GTT CCC CAT ATC CAG TG-3 and reverse, 5-ATC TGC TGA GTC TCC CAG TG-3; human PAFR: forward, 5-CCT CCT TAG CAC CAA CTG TGT C-3 and reverse, 5-CAA CCA CTT CAG TGA CCG TAT CC-3; human RORC2: forward, 5-CAG TCA TGA GAA CAC AAA TTG AAG TG-3 and reverse, 5-CAG GTG ATA ACC CCG TAG TGG AT-3. Each sample for the real-time PCR consisted of: 1 *μ*L cDNA, 2.5 mM MgCl_2_, 100 *μ*M dNTP, 1 *μ*M of primers, 2.5 *μ*L of 10X PCR buffer, 0.5 unit of Taq polymerase (Feldan Bio Laboratories Inc, Québec, QC, Canada) and 0.8 *μ*L of SYBR Green (Molecular Probe, Eugene, OR; 1/1000 stock dilution) in a reaction volume of 25 *μ*L. The cycling program consisted of an initial denaturation at 95°C for 5 min, 45 cycles of amplification conditions as follows: 95°C (30 sec), 58°C (30 sec), 72°C (30 sec), and a final extension at 72°C for 6 min. Each gene expression was normalized with GAPDH mRNA content and fold differences were calculated with the delta-delta (ΔΔ)Ct method according to the following formula: (ΔΔCt = [(Ct G.O.I.Ctl − Ct HK.G.Ctl) − (Ct G.O.I.STIM. − Ct HK.G.STIM.)]. Comparison of the expression of each gene between its control and stimulated states was determined by its ΔΔCt. Results were then transformed into fold variation measurements: fold increase = 2^ΔΔCt^

### 2.7. Statistical Analysis

Statistical significance was calculated using Prism 5 software (GraphPad Software, San Diego Calif, USA). For analysis of differences between experimental groups, Student's *t*-test and one-way and two-way ANOVA with Bonferroni posttest were used, as appropriate. Values of *P* ≤ 0.05 were considered statistically significant.

## 3. Results

### 3.1. PAF Induces IL-23, IL-6, and IL-1*β* Production

**Figure 1:**
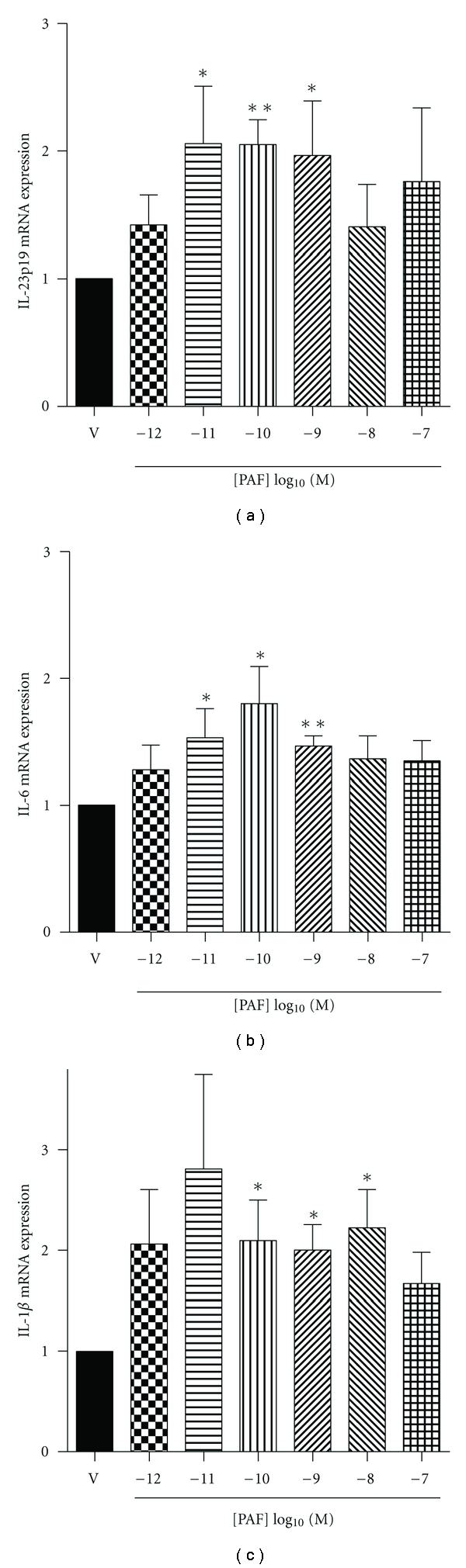
PAF-induced IL-6 and IL-23p19 mRNA expression in LC. Monocyte-derived LC were stimulated with graded concentrations of PAF or its vehicle (ethanol; V) for 4 h. IL-23 p19 (a), IL-6 (b), and IL-1*β* (c) mRNA was then measured by real-time quantitative PCR. Data (means ± SEM) are expressed as fold induction relative to vehicle control. *n* = 9; *P < 0.05; **P < 0.01.

In order to assess the potential for PAF to modulate Th17 cell development, we initially exposed monocyte-derived LC to graded concentrations of PAF and measured their capacity to express IL-23p19, IL-6, and IL-1*β* mRNA. As shown in Figure 1, picomolar concentrations of PAF increased both IL-23, IL-6 and IL-1*β* gene expression in a 4-hr culture, with significant effects at PAF concentrations of 10^−11^ to 10^−9^ M.

**Figure 2:**
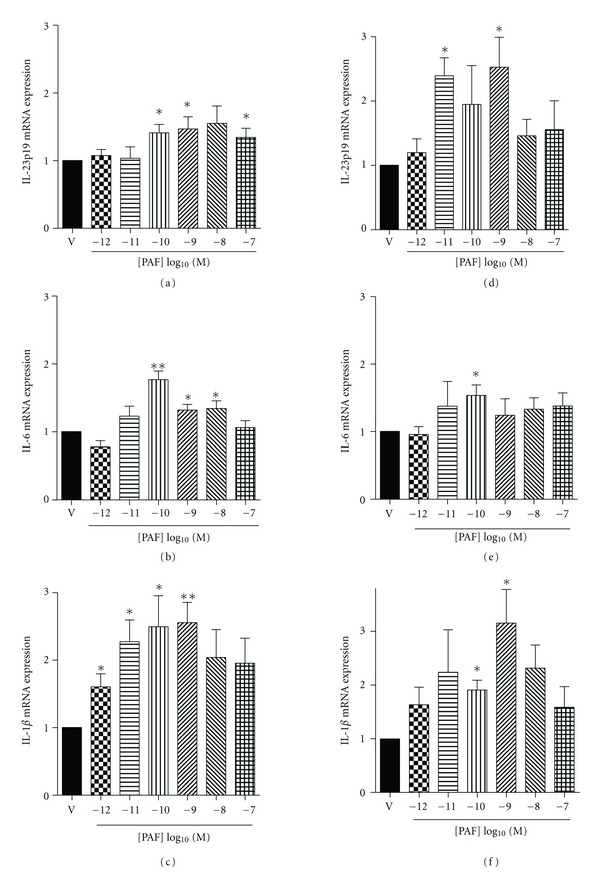
PAF-induced IL-23p19, IL-6, and IL-1*β* mRNA expression in A431 keratinocytic cells (a, b, c) and normal human epidermal keratinocytes (NHEK; d, e, f). Cells were stimulated with graded concentrations of PAF or its vehicle (ethanol; V) for 4 h. IL-23 p19 (a, d), IL-6 (b, e), and IL-1*β* (c, f) mRNA was then measured by real-time quantitative PCR. Data (means ± SEM) are expressed as fold induction relative to vehicle control. *n* = 8; *P < 0.05; **P < 0.01.

Since keratinocytes also express receptors for PAF (PAFR) [22], we tested whether PAF could also induce cytokine expression in these cells. As shown in Figure 2, PAF induced the expression of IL-23p19, IL-6 and IL-1*β* mRNA in both A431 keratinocytic cells (Figures 2(a), 2(b), and 2(c)) and normal human epidermal keratinocytes (NHEK) (Figures 2(d), 2(e), and 2(f)) with significant increases at PAF 10^−10^ to 10^−8^ M.

### 3.2. PAF-Activated LC Induce Th17 Cell Development

Since antigen-presenting cells, such as LC can modulate T helper cell polarization, we investigated whether PAF activation of LC could induce the development of a Th17 phenotype in cocultured autologous CD4^+^ T cells. During the 5-day differentiation of monocytes into LC, we activated autologous T cells with anti-CD3 and anti-CD28 Ab. Monocyte-derived LC expressed both PAFR mRNA and protein, and were capable of responding to PAF with calcium flux and chemotaxis. In contrast, activated CD4^+^ T cells expressed no PAFR mRNA and failed to respond to PAF in either calcium flux or chemotaxis. 

**Figure 3:**
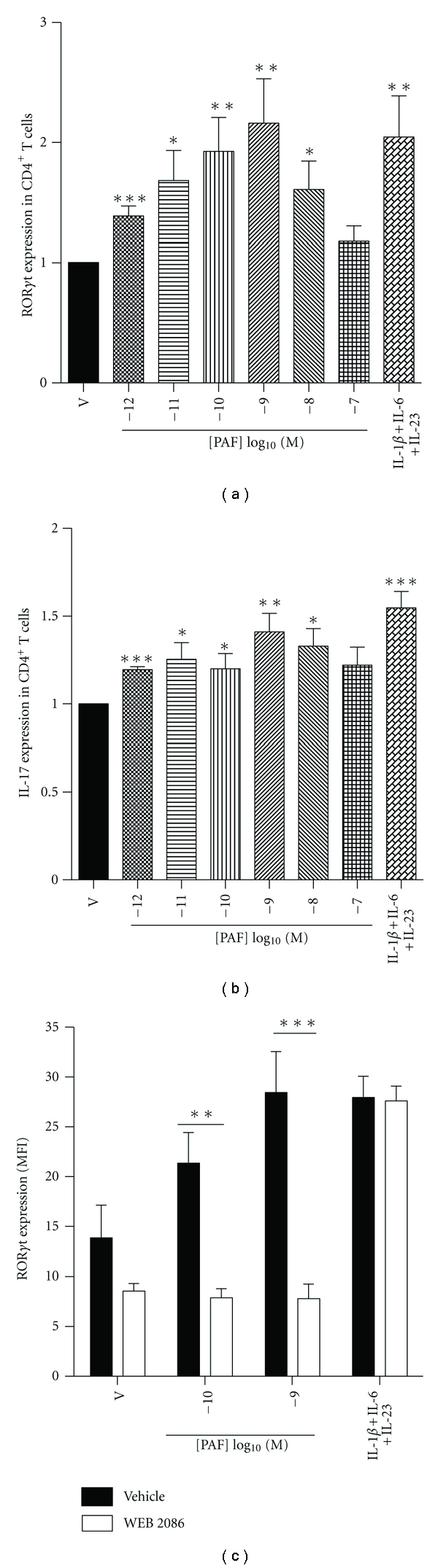
PAF-stimulated LC induce a Th17 phenotype in cocultured T cells. Monocyte-derived LC were stimulated with graded concentrations of PAF or its vehicle (ethanol; V) and cocultured with antiCD3/CD28-activated CD4^+^ T cells for 5 days. ROR*γ*t (a) and IL-17 (b) expression was measured by FACS in CD4^+^ T cells. For comparison, CD4^+^ T cells were cultured alone with IL-1*β*, IL-6, and IL-23 for 5 days. Data (means ± SEM) are expressed as fold induction relative to vehicle (V) control. *n* = 10; *P < 0.05; **P < 0.01; ***P < 0.001. (c) LC were also stimulated with either vehicle or PAF in the absence or presence of the PAFR antagonist WEB 2086 (10^−5^ M) and cocultured with anti-CD3/CD28-activated CD4^+^ T cells for 5 days. ROR*γ*t expression was measured by FACS in CD4^+^ T cells and expressed as geometric mean (±SEM) fluorescence intensity (MFI). *n* = 5; **P < 0.01; ***P < 0.001.

Following their 5-day differentiation, LC were treated with graded concentrations of PAF and put in a coculture with activated autologous T cells for an additional 5-day period at a ratio of 1 : 5. T cells were then analyzed by flow cytometry for expression of the transcriptional regulator ROR*γ*t and for IL-17. As shown in Figures 3(a) and 3(b), PAF-activated LC significantly enhanced both ROR*γ*t and IL-17 expression in preactivated and cocultured CD4^+^ T cells. Induction of ROR*γ*t expression in T cells by PAF-pretreated LC was of a similar magnitude as that induced in activated T cells cultured in the presence of IL-1*β*, IL-6, and IL-23 without LC. As expected, PAF-induced ROR*γ*t expression in T cells was prevented by pretreatment of LC with the PAFR antagonist WEB2086 before exposure to PAF, whereas cytokine-induced ROR*γ*t expression was unaffected (Figure 2(c)). Interestingly, exposure of LC to WEB2086 lowered their basal level of ROR*γ*t induction, suggesting that basal ROR*γ*t expression was dependent on PAFR activation, potentially by endogenous production of PAF or PAF-like compounds by LC.

### 3.3. PAF-Induced Th17 Development Is Associated with Enhanced Production of IL-17A, IL-21, IL-22, and RORC2

**Figure 4:**
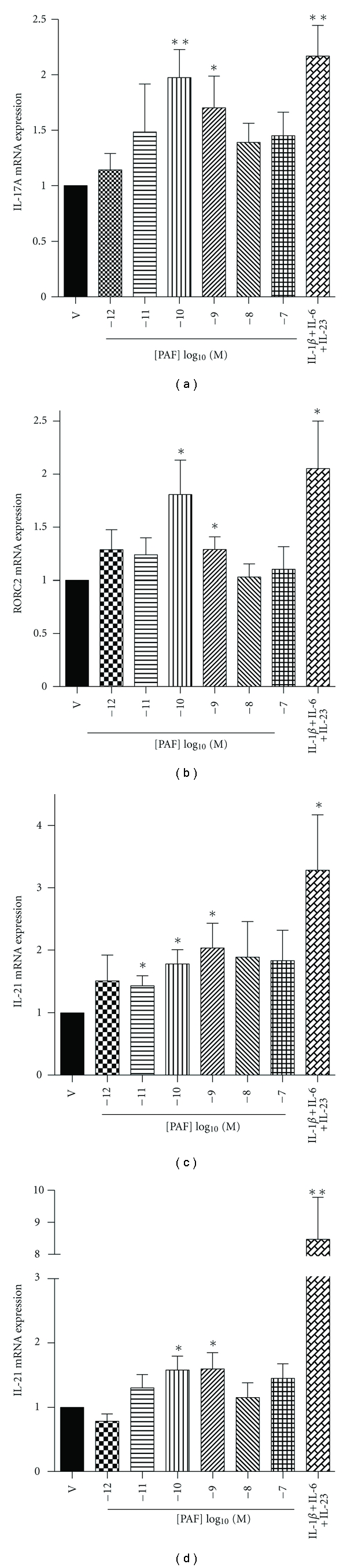
PAF-stimulated LC induce Th17 mRNA markers in cocultured T cells. Monocyte-derived LC were stimulated with graded concentrations of PAF or its vehicle (ethanol; V) and cocultured with antiCD3/CD28-activated CD4^+^ T cells for 5 days. IL-17A (a), RORC2 (b), IL-21 (c), and IL-22 (d) mRNA expression was measured by real-time quantitative PCR in CD4^+^ T cells. For comparison, CD4^+^ T cells were cultured alone with IL-1*β*, IL-6, and IL-23 for 5 days. Data (means ± SEM) are expressed as fold induction relative to vehicle (V) control. *n* = 8; *P < 0.05; **P < 0.01.

Th17 development following coculture with PAF-treated LC was accompanied by expression of Th17-associated cytokines by the CD4^+^ T cell population, namely IL-17A, IL-21, and IL-22, as well as RORC2, the closest human homologue of mouse ROR*γ*t. As shown in Figure 4, subnanomolar concentrations of PAF were able to induce a significant increase in IL-17A, IL-21, IL-22, and RORC2 mRNA expression. Interestingly, the PAF-induced LC-dependent levels were similar to those induced by direct T cell activation with the combination of IL-1*β*, IL-6, and IL-23, except for IL-22, which was expressed more strongly in directly activated T cells. In contrast, purified CD4^+^ T cells, either freshly isolated or activated for 5 days with anti-CD3/anti-CD28 Ab, failed to upregulate their expression of IL-17A or RORC2 in response to PAF (data not shown).

### 3.4. PAF-Induced Th17 Development Is Dependent on IL-23 and IL-6

**Figure 5:**
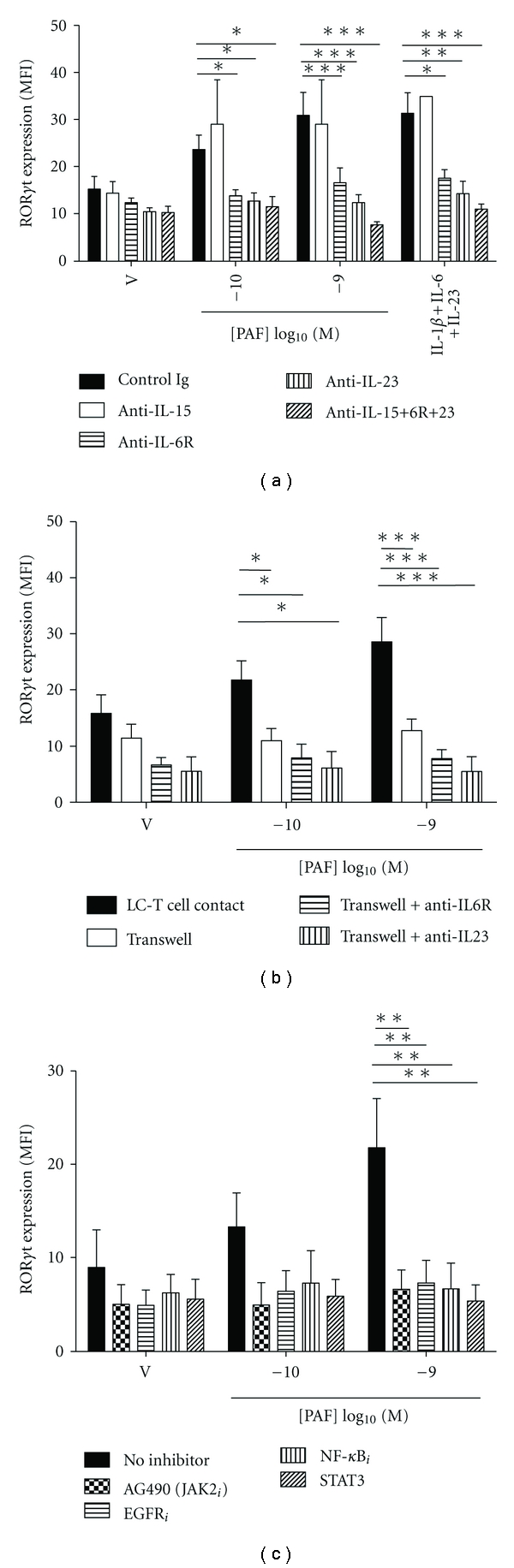
PAF-stimulated Th17 development is dependent on cytokines, LC-T cell contact and selected signaling pathways. (a) Monocyte-derived LC were stimulated with either vehicle or PAF in the presence of neutralizing Ab to IL-15, IL-6R, and/or IL-23, or control Ig, and cocultured with antiCD3/CD28-activated CD4^+^ T cells for 5 days. For comparison, CD4^+^ T cells were cultured alone with IL-1*β*, IL-6 and IL-23 for 5 days in the absence or presence of the above-indicated neutralizing Ab. ROR*γ*t expression was measured by FACS in CD4^+^ T cells and expressed as geometric mean (±SEM) fluorescence intensity (MFI). *n* = 5; *P < 0.05; **P < 0.01; ***P < 0.001. (b) LC were stimulated with either vehicle or PAF and cocultured with antiCD3/CD28-activated CD4^+^ T cells for 5 days with direct LC-T cell contact or separated by a Transwell filter (LC in the top chamber, T cells in the bottom), in the absence or presence of neutralizing Ab to IL-6R or IL-23. ROR*γ*t expression was measured by FACS in CD4^+^ T cells and expressed as geometric mean (±SEM) fluorescence intensity (MFI). *n* = 5; *P < 0.05; ***P < 0.001. (c) LC were stimulated with either vehicle or PAF and cocultured with antiCD3/CD28-activated CD4^+^ T cells for 5 days in the absence or presence of inhibitors of Jak2, EGFR, NF-*κ*B, or STAT3. ROR*γ*t expression was measured by FACS in CD4^+^ T cells and expressed as geometric mean (±SEM) fluorescence intensity (MFI). *n* = 4; **P < 0.01; ***P < 0.001.

We next investigated whether PAF-induced development of Th17 differentiation was dependent on cytokines produced by LC. Since IL-15, a proinflammatory cytokine, synthesized mainly by LC and DC, has also been suggested as an inducer of Th17 cell development [33, 34], we also used neutralizing anti-IL-15 Ab in our assays. We thus added neutralizing anti-IL-6R, anti-IL-23 and/or anti-IL-15 Ab to the LC-T cell cocultures and measured ROR*γ*t expression in CD4^+^ T cells after 5 days. As shown in Figure 5(a), addition of Ab directed against IL-23 or IL-6R resulted in a markedly decreased induction of ROR*γ*t expression. In contrast, neutralization of IL-15 had no effect. Our data suggest that IL-23 and IL-6, but not IL-15, are essential for LC-dependent, PAF-induced Th17 development.

### 3.5. PAF-Induced Th17 Development Is Dependent on LC-T Cell Contact

Since antigen-presenting cells need to physically interact with T cells at different stages of the immune response, we tested whether cell-cell contact between PAF-primed LC and TCR-activated T cells was essential for Th17 development. As shown in Figure 5(b), ROR*γ*t expression in CD4^+^ T cells was prevented when LC were separated from T cells by a Transwell membrane, indicating that indeed PAF-induced Th17 development was also dependent on LC-T cell contact.

### 3.6. PAF-Induced Th17 Development Is Dependent on Jak2, STAT3, NF-*κ*B, and EGFR

We have previously shown that PAFR signaling could induce Jak2 phosphorylation and STAT3 translocation in monocytes [35]. Moreover, PAF had been shown to induce the production of heparin-binding epidermal growth factor like growth factor (HB-EGF) through an NF-*κ*B-dependent mechanism [36] and to transactivate the epidermal growth factor receptor (EGFR) [37]. STAT3 and NF-*κ*B were also found to be required for IL-23 mediated IL-17 production [38]. When LC were pretreated with cell-permeable inhibitors of Jak2, EGFR, NF-*κ*B or STAT3, ROR*γ*t expression in CD4^+^ T cells was totally prevented (Figure 5(c)), suggesting that the Jak-STAT, NF-*κ*B and EGFR signaling pathways were involved in PAF-induced Th17 development.

## 4. Discussion

The expression of IL-23 is highly enhanced in lesional psoriatic skin and this Th17-inducing factor is produced by keratinocytes, Langerhans cells, dermal dendritic cells and macrophages [19]. The immunogenicity of skin correlates with a substantial number of resident DCs including epidermal Langerhans cells (LCs) and dermal DCs (DDCs), which are both capable of activating naïve T cells [39, 40]. During the development of chronic cutaneous inflammatory and autoimmune disorders, such as in psoriasis, Th17 and Th1 cells infiltrate the skin where resident DCs were shown to have a role in the initiation and maintenance of Th17 immunity [41, 42]. Different observations suggested a possible association of the inflammatory lipid mediator PAF with psoriasis [29–32]. PAF is found in increased concentrations in inflammatory lesions and is a known regulator of transcription of a variety of cytokines [26] including IL-6. Moreover, PAF appears very early in inflammation and can stimulate and be produced by a variety of cells such as monocytes, dendritic cells, and keratinocytes. In this context, we investigated whether PAF could affect the development of Th17 cells in a model of LC-T cell coculture. 

In the present study we provide evidence that PAF could effectively modulate the development of Th17 cells. First, we found that PAF induced a rapid expression of IL-1*β*, IL-6 and IL-23 in human LC and keratinocytes, three cytokines reported to be crucial for Th17 development [1, 2]. Second, we observed that TCR-activated T cells in coculture with PAF-stimulated LC, developed a Th17 phenotype with increased expression of the transcriptional regulator ROR*γ*t (and its human RORC2 mRNA equivalent) and associated molecules including IL-17A, IL-21 and IL-22. Third, our findings demonstrate that blocking PAFR in LC-T cell cocultures impaired the expression of the transcriptional regulator ROR*γ*t and that using anti-IL-6R and anti-IL-23 alone or in combination, abrogated the induction of ROR*γ*t expression by PAF. Finally, we also found that PAF-induced Th17 development was dependent on cell-cell contact between LC and T cells and that PAF enhanced the expression of Th17-associated markers via stimulation of LC rather than T-cells. Taken together, these observations suggest that generation of Th17 by LC is dependent on T cell interaction with LC and the production, by the latter, of cytokines such as IL-6 and IL-23 in response to PAF. 

This kind of regulation of IL-17 production was also reported for another G-protein-coupled receptor. Sphingosine 1-phosphate (S1P), a biologically active lysophospholipid that binds to its receptor S1P_1_, was shown to have an effect similar to IL-23 in terms of increasing Th17 development from mouse splenic CD4^+^ T cells [43]. Moreover, in a recent review article, Edwards and Constantinescu suggested that the PAF/PAFR pathway may directly influence T cell responses and favour a Th17 phenotype, but no actual data were provided [28]. There are conflicting observations about whether human T lymphocytes express PAFR or not. Some authors reported that PAFR was not detectable on either resting [44] or activated T cells [45], but others observed low level expression on resting T cells which increased following stimulation [28, 44]. However, in our model, TCR-activated highly purified human CD4^+^ T lymphocytes lacked PAFR expression and were unable to functionally respond to PAF stimulation. On the other hand, our results demonstrating that PAF can stimulate human LC and keratinocytes are in concordance with the literature which indicates that both DC and keratinocytes express functional PAFR [22, 46] and that PAF signalling activates LC migration [47]. As seen in our results, a bell-shaped concentration-response curve is regularly seen with GPCR ligands, where high concentrations do not elicit as good a response as mid-range concentrations. It is thought that autologous desensitization and internalization of the receptor, in response to high ligand concentrations, may be responsible for this phenomenon.

IL-17A and IL-17F both induce the production of various cytokines and chemokines, including TNF*α*, IL-1*β*, IL-6, IL-8, CCL2 (MCP-1), granulocyte colony-stimulating factor (G-CSF), as well as the expression of intercellular adhesion molecule (ICAM)-1 by monocytes, airway epithelial cells, vein endothelial cells, and fibroblasts. These IL-17-induced expression profiles can often be enhanced by TNF*α* and IFN-*γ* [48]. In the context of the skin, IL-17 has been reported to modulate the cytokine production and surface molecular make-up of epidermal keratinocytes. IL-17 enhanced the production of IL-6 and IL-8 in keratinocytes and induced a weak expression of ICAM-1 and HLA-DR [41], whereas IFN-*γ* and TNF*α*-induced production of RANTES was markedly inhibited by IL-17 [49]. In addition, IL-17 has been reported to modulate fibroblast function by inducing their production of IL-6, IL-8, IL-11, GRO*α*, and G-CSF [48]. 

In addition to IL-17A and IL-17F, Th17 cells produce other effector cytokines, namely, IL-21 and IL-22 [6]. Neither IL-21 nor IL-22 are Th17-exclusive cytokines, but are preferentially expressed in Th17 cells. IL-21 is produced mainly by CD4^+^ T cells [50] as well as by NKT cells [51]. Recently, several groups simultaneously observed that IL-21 was produced by Th17 cells upon stimulation by IL-6 and by IL-21 itself and exerted critical functions in Th17 cell development [52–54]. The coexpression of IL-17 and IL-22 in Th17 cells suggests that the pathways that regulate these two cytokines might be very similar [55]. Although IL-23 is insufficient to induce *de novo* IL-17 production from naïve CD4^+^ T cells, IL-23 alone promotes IL-22 production from many different immune cell types. IL-6 alone is sufficient for the induction of IL-22 from naïve CD4^+^ T cells. IL-22, therefore, might be an obvious downstream factor of IL-23 that mediates the crosstalk between infiltrating immune cells, especially T cells, and keratinocytes in psoriatic skin. Injection of IL-23 into a mouse ear causes an inflammatory skin phenotype, characterized by leukocyte infiltration [54]. The infiltrating CD4^+^ T cells display a Th17 cell phenotype with the expression of both IL-17 and IL-22. In addition to IL-21 and IL-22, other cytokines such as TNF*α* and IL-1*β*, which are not specifically produced by Th17 cells, have been proposed to have an additional role in the amplification of Th17 responses [56, 57]. PAF, a known activator of IL-1*β* and TNF*α* [58] could also participate in such an amplification loop. Recently, TLR2-activated human LC also were shown to promote the polarization of Th cells into Th17 cells via the production of IL-1*β*, TGF*β*, and IL-23 [59].

Thus, PAF/PAF-R interactions may be involved in the early events leading to Th17 differentiation and trigger a self-amplification process. PAF may contribute to the creation of a proinflammatory environment that would skew cytokine production in favour of Th17 development. During the preparation of the present paper, Singh et al. [60] reported that *in vivo *blockade of PAF inhibited the development of psoriasis-like disease in a transgenic mouse model and the concomitant Th17 phenotype.

## 5. Conclusion

In the present work, we showed that PAF stimulates LC and keratinocytes to produce IL-6 and IL-23. Moreover, activated T cells in coculture with PAF-stimulated LC develop a Th17 phenotype with increased expression of the transcriptional regulator ROR*γ*t and enhanced production of IL-17, IL-21 and IL-22. These effects are blocked by a PAF receptor antagonist and neutralizing antibodies to IL-6 and IL-23. These observations suggest that PAF can contribute to the differentiation of activated T cells into Th17 by inducing LC to produce IL-6 and IL-23. This may constitute a previously unknown stimulus for the development and persistence of inflammatory processes that could be amenable to pharmacologic intervention.

## Abbreviations

LC: Langerhans cell
PAF: Platelet-activating factor
PAFR: PAF receptor
ROR*γ*t: Retinoic orphan receptor-gamma T
RORC2: Retinoic orphan receptor C2.
